# Integrating T cell signaling and metabolism to enhance T cell engager responses in solid tumors

**DOI:** 10.3389/fimmu.2026.1887842

**Published:** 2026-07-22

**Authors:** Michael Scaglione, Minal Engavale, Matthew G Chun, Deepali V Sawant, Daniel R Lu, Edward LaGory

**Affiliations:** Amgen R&D Postdoctoral Fellows Program, South San Francisco, CA, United States

**Keywords:** immunotherapy, metabolism, signaling, solid tumors, T cell engagers (TCEs), tumor microenvironment (TME)

## Abstract

T cell engagers (TCEs) have delivered meaningful clinical benefit to patients, with eight molecules currently FDA-approved for hematologic malignancies and two approved for solid tumor indications. Despite their transformative potential, successful TCE development across solid tumor indications remains challenging, and additional strategies are needed to maintain T cell fitness and function within the tumor microenvironment (TME). Next-generation TCE designs aim to increase response rate and bolster durability by optimizing or delivering additional signals to T cells. In recent years, cellular metabolism has emerged as a potent regulator of T cell function and fate, shaping immunity by supporting the biochemical requirements of immunological effector functions and acting as a direct immunoregulatory signal from the TME itself. Despite this, neither cell-intrinsic nor environmental roles for metabolism in regulating TCE responses in solid tumors have been explicitly explored. We propose that metabolism is a powerful lens for understanding TCE efficacy and resistance in solid tumors, integrating signals from both surface receptors and the biochemical environment of the TME to shape T cell function and therapeutic response. In this mini-review, we highlight how three classical T cell signaling axes - 1) the T cell receptor complex, 2) costimulatory receptors, and 3) cytokine receptors – drive metabolic rewiring to license immune function and shape T cell fate. We also explore how environmental cues such as nutrients or metabolic stressors govern T cell responses, highlighting how biochemical perturbations within the TME could hamper TCE efficacy. Finally, we highlight emerging methods for dissecting metabolic contributions to TCE responses, proposing that understanding the interplay between immunological signaling, cellular metabolism, and immune programming could inform the design of next-generation TCEs for solid tumors.

## Introduction

T cell engagers (TCEs) are emerging as a new therapeutic approach that redirect a patient’s endogenous T cells to kill cancer cells. They do so by simultaneously engaging a tumor-associated antigen (TAA) expressed on the surface of cancer cells and CD3 expressed by the T cells. TCEs trigger T cell receptor (TCR) signaling and T-cell-mediated tumor killing independent of antigenic peptide presentation by major histocompatibility complex molecules. Clinical proof of concept for TCEs was first demonstrated with the anti-CD19 BiTE^®^ (bispecific T-cell engager) molecule blinatumomab, which is approved by the FDA for the treatment of CD19-positive Philadelphia chromosome-negative B-cell precursor acute lymphoblastic leukemia (1). The clinical success of blinatumomab motivated the development of numerous TCEs for hematologic and solid cancers (2), with two TCEs in the last four years receiving FDA-approval for use in solid tumor indications – tebentafusp for uveal melanoma (3, 4) and tarlatamab for small cell lung cancer (5, 6). Over 100 clinical trials evaluating the safety and efficacy of TCEs have been initiated in the last decade (7–10), underscoring the intense interest in this modality.

However, TCE development in solid tumors remains challenging, both because of the expression profile of tumor antigens and because of the tumor microenvironment (TME). Redirected T cells must function within an immunosuppressive TME (11–14), where hypoxia, nutrient deprivation, suppressive myeloid and regulatory T cells, and stromal barriers limit T cell infiltration and effector function. Additionally, chronic CD3-driven stimulation in this setting can drive TCE-redirected T cells toward exhaustion-like, dysfunctional states (15–18). Together, these pressures imposed by the solid TME erode T cell fitness and persistence, undermining durable and efficacious TCE responses. This suggests that next-generation solid-tumor TCEs should be designed not only to trigger acute tumor killing, but also to support durable immune responses by maintaining T cell fitness and function despite the immunosuppressive and metabolically restrictive conditions associated with the TME.

Our understanding of therapeutic immune responses is founded on decades of investigation into the factors governing lymphocyte activation and function. Since the development of the “two-signal” model by Bretcher and Cohn in 1970 (19, 20), our molecular understanding of the signals governing T cell function and fate has grown immensely, with a model largely focusing on three signaling axes: 1) the T cell receptor (TCR) signaling complex, 2) costimulatory receptor signaling, and 3) signaling via cytokines (21, 22). However, in the last two decades, cellular metabolism has emerged as essential for productive T cell responses, acting downstream of surface-receptor signaling to meet the energetic and biosynthetic requirements for productive T cell activation and protective immunity (23). Additionally, recent evidence suggests that environmental biochemical factors act as an additional “Signal 4’ to T cells, with nutrients and metabolic stressors promoting or dampening anti-tumor immunity, respectively (24). While the importance of T cell metabolism has had a large impact on chimeric antigen receptor T cell (CAR-T) development (25–27), its role in TCE responses is less explored.

In this review, we focus on metabolism as a regulator of T cell biology, highlighting how classical surface receptor signaling and environmental programming jointly govern T cell responses via metabolic rewiring and exploring its implications for understanding TCE pharmacodynamic response and efficacy in the solid tumor setting. Additionally, we propose that a deeper understanding of the intersection between signaling, metabolism, and T cell function during TCE response is needed to inform the rational design of next-generation TCE candidates that optimize T cell metabolic and functional capacity for sustained cytolytic activity.

## Signal 1 – TCR/CD3 signaling and metabolic rewiring in early T cell activation, recall, and programming

**Figure 1:**
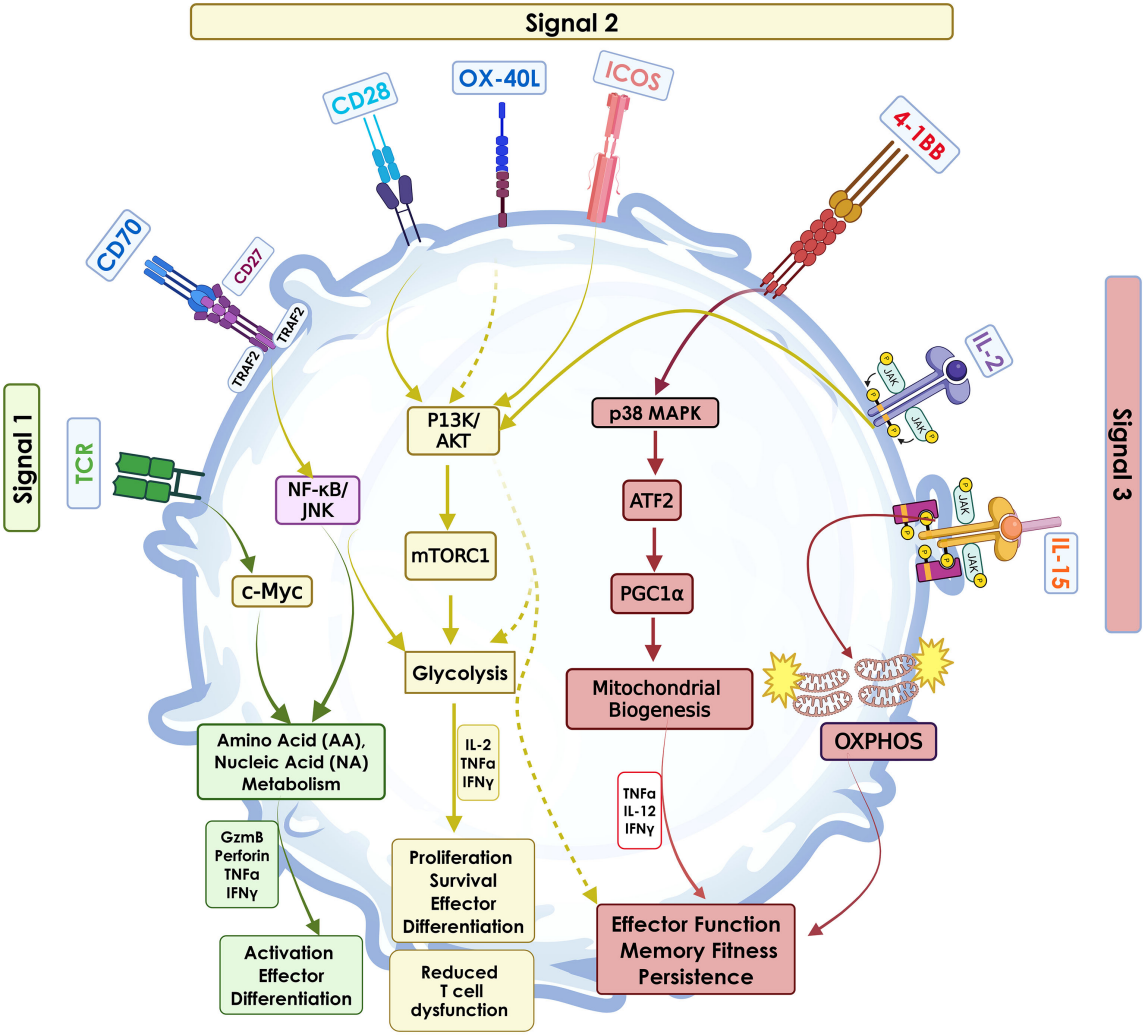
Signals 1–3 converge on metabolic programming to shape T-cell fate, fitness, and antitumor function. Schematic illustrating how Signal 1, Signal 2, and Signal 3 integrate with intracellular metabolic programs to regulate T-cell activation, differentiation, effector function, and persistence. Signal 1 through the T-cell receptor (TCR) initiates activation-associated metabolic reprogramming, including c-Myc-driven anabolic pathways that support amino acid and nucleic acid metabolism and early effector differentiation. Signal 2, delivered by costimulatory receptors including CD28, OX40, ICOS, 4-1BB, and CD27, reinforces PI3K/AKT/mTORC1, NF-kappaB/JNK, glycolytic, and mitochondrial programs that promote proliferation, survival, cytokine production, and sustained functional capacity.Signal 3 cytokines, represented here by IL-2 and IL-15, further tune these responses by supporting either glycolytic effector output or OXPHOS- and mitochondrial biogenesis-associated memory fitness and persistence. Together, these coordinated signaling-metabolic circuits enhance antitumor function, reduce T-cell dysfunction, and support durable responses in T-cell engager and CAR T-cell therapies. Created in BioRender. Engavale, M. (2026) https://BioRender.com/6312gh2.

T cell responses are initiated by engagement of the TCR signaling complex, leading to immune synapse formation, T cell activation, and cytotoxic function and/or cytokine release (**Figure 1**) (28). Recent studies have defined that metabolic reprogramming is integral to productive T cell activation (23). Upon TCR engagement, T cells rapidly engage glycolysis and glutaminolysis pathways, increase nutrient import and ATP production, and drive macromolecule biosynthesis to support the demands of cell proliferation and function (29–39). This process of metabolic rewiring is both biochemical and genetic in nature, coordinated by signaling and transcriptional pathways such as mammalian target of rapamycin complex 1 (mTORC1), c-Myc, and IRF4 that license activation and proliferation by reprogramming transcriptional and biosynthetic state (31, 32, 34–36, 40, 41). In lieu of modality-specific investigation, these studies suggest that T cell metabolism could play an important role in mediating TCE responses by promoting productive T cell activation.

T cell subsets differ in their functional responses to TCR signaling, creating opportunities to shape TCE therapeutic response by targeting subsets with favorable profiles (42, 43). In particular, memory T cells have been identified as potential primary mediators of TCE response (15, 44, 45), given their increased sensitivity to TCR signaling, less strict requirements for costimulation, and ability to mount rapid and potent effector responses upon recall (46–54). While less is known about the metabolic requirements specific to memory recall responses, emerging studies suggest that differences in glycolysis (55, 56), acetate handling (57), and the ability to store and use glycogen (58–60) provide memory cells with biochemical advantages that underlie their unique protective functionality.

The affinity of the TCR:peptide-MHC interaction governs endogenous T cell immune responses by shaping clonal expansion and cell differentiation (61). Similarly, TCE anti-CD3 binding affinity impacts the strength of T cell signaling and shapes *in vitro* TCE therapeutic window by dampening cytokine production relative to cytotoxicity (62–67). Interestingly, NAD biosynthesis and glycolytic activity are particularly sensitive to TCR signal strength, correlating with the downstream expression of c-Myc and IRF4 and directly regulating long-term cell proliferation potential (29, 40, 41). Additionally, the kinase ITK is proposed to modulate perceived signal strength and immune function after TCR engagement, in part via its activity on metabolic programming (68, 69). Given that both affinity-tuning and ITK inhibition by ibrutinib are currently being evaluated to improve the therapeutic window of T cell therapies (70, 71), these data suggest that understanding the relationship between TCE anti-CD3 affinity, proximal TCR signaling, and metabolic activity could help explain differences in functional responses across TCE candidates or reveal additional opportunities to optimize TCE safety or efficacy.

Lastly, chronic TCR signaling drives T cell exhaustion, a progressive differentiation process that ultimately leads to T cell dysfunction and limits the activity and durability of T cell therapies including TCEs (72–74). Further, providing intermittent rest from TCR signaling has been shown to mitigate exhaustion and improve the efficacy of T cell-targeted therapies (75, 76). Given that T cell exhaustion is both associated with metabolic deficits and influenced by biochemical stressors in the TME (discussed in “Signal 4”), future work could help dissect how synergy between environmental stressors and the metabolic demand driven by chronic TCR signaling influence exhaustion and regulate T cell fate during TCE responses (77–81).

## Signal 2 – costimulation as metabolic licensing for durability

Costimulatory signaling or signal 2 (**Figure 1**) is a central component of physiological T-cell activation, providing secondary cues beyond TCR engagement that support activation, expansion, and differentiation while preventing anergy (82–84). In TCEs, however, T cells are redirected to tumor cells rather than professional APCs and may not receive the full complement of costimulatory signals provided during physiological priming (85, 86). These inputs are mediated by multiple receptor families, including immunoglobulin superfamily members such as CD28 and ICOS (Inducible T-cell Costimulator, CD278) (87), and the tumor necrosis factor receptor (TNFR) superfamily, which includes CD27, OX40, and 4-1BB (83, 88, 89). Collectively, these receptors do not merely augment activation but shape T-cell fate by directing proliferation, survival, differentiation, and long-term persistence (90, 91). Notably, each costimulatory pathway is coupled to distinct downstream signaling and metabolic circuits, which in turn influence the efficiency of T cells to sustain effector responses and persist in demanding environments (26, 92–95).

Among these, CD28 and 4-1BB illustrate how costimulatory inputs can differentially program T-cell metabolism and function (25, 26). CD28 augments phosphatidylinositol 3-kinase PI3K/Akt/mTORC1 signaling increasing glucose uptake and glycolytic flux to support the anabolic demands of proliferation, cytokine production, and effector function (94, 96–98). CD28 signaling also primes mitochondria early after activation by promoting fatty-acid oxidation and remodeling mitochondrial cristae, building latent respiratory reserve for later rechallenge (99). By contrast, 4-1BB is induced after activation and its signaling drives mitochondrial biogenesis, fusion, and increased respiratory capacity by inducing peroxisome proliferator-activated receptor gamma coactivator 1-alpha (PGC1a) (95, 100–103). These distinct metabolic programs may be particularly relevant in solid tumors, where the TME may repress mitochondrial biogenesis and drive intratumoral metabolic insufficiency and dysfunction (95, 103, 104). In CAR-T cells, these metabolic programs align with distinct cell states, with CD28-driven signaling favoring glycolytic, effector or effector-memory differentiation and 4-1BB favoring mitochondrial fitness, central-memory-like differentiation, and persistence (25, 26, 104, 105). These studies suggest that TCEs incorporating CD28 or 4-1BB signaling may differentially program redirected T cells, thereby enhancing killing and durable antitumor responses (106). Thus, the critical question for TCE design may not be whether one costimulatory program is inherently superior, but how CD28 and 4-1BB-driven states can be matched to distinct phases of the redirected T-cell response to promote durable antitumor activity, a possibility that now warrants broader preclinical evaluation.

Beyond CD28 and 4-1BB, additional costimulatory receptors are being investigated as ways to tune TCE responses (107–109). In this context, OX40 supports survival and bioenergetic capacity (92, 109), CD27 reinforces anabolic metabolism and memory-associated programming (93, 110), ICOS promotes metabolic fitness by reducing glucose dependencies (111), and CD2-CD58 enhances synapse quality and signaling efficiency (112, 113). Most trispecific TCE strategies have centered on CD28 or 4-1BB costimulatory modules, with CD2 gaining interest more recently. For eg, a trispecific PSMAxCD3xOX40 construct, compared with a bispecific PSMAxCD3, enhanced antitumor activity in a prostate cancer model while increasing both glycolysis and oxidative phosphorylation (107). Similarly, trispecific TCEs incorporating CD58 to deliver integrated CD2 costimulation improved T-cell viability and effector function under chronic stimulation (114). Although the metabolic consequences of this approach have not yet been defined, an important next question is whether improved CD2-CD58 engagement also helps preserve metabolic fitness during prolonged antigen exposure.

Together, these studies suggest that alternative costimulatory support can improve TCE efficacy not simply by augmenting CD3 signaling but by directing T cells toward distinct functional states in concert with Signal 1, with consequences for metabolic fitness, function and persistence (107). For next-generation TCEs, the design opportunity is therefore to pair an appropriate CD3 input with matched costimulatory support, so that signal strength, metabolic plasticity, and persistence are tuned together rather than optimized in isolation.

## Signal 3 – cytokines as metabolic enablers of redirecting T-cell therapies in solid tumors

Even when T-cell therapies including TCEs and CAR-T’s incorporate costimulatory input, cytokine-driven Signal 3 remains necessary for full effector differentiation and durable function (**Figure 1**) (115–117). In solid tumors, this need is amplified because continuous anti-CD3 stimulation can drive exhaustion, making cytokine support important for preserving the metabolic fitness required for proliferation, persistence, and serial killing (115, 118–121). Among the common gamma chain (γc) family of cytokines, IL-2 and IL-15 are the principal cytokines currently being evaluated in cancer immunotherapy and adoptive T-cell settings (122). Both signal through IL-2/15Rβ (CD122) and γc (CD132), activating JAK1/JAK3-STAT5 with additional PI3K-AKT-mTOR and MAPK/ERK signaling that links proliferation and survival to metabolic and cytotoxic programming (123, 124). This mechanistic framework is beginning to inform preclinical T-cell therapy design, where IL-2- and IL-15-based strategies are being explored to improve the quality, durability, and antitumor activity of redirected T-cell responses, albeit with the limitations inherent to preclinical models. In the context of TCEs, local co-delivery of IL-2 with a MUC1×CD3 engager increased granzyme B, perforin, and interferon gamma, reduced exhausted CD8 phenotypes, and improved tumor control (125).

A more controlled approach is gated IL-2 delivery, as shown by IL-2-armored pMHC-IgG engagers, which expand effector-memory CD8+ T cells and promote tumor-cell cytotoxicity with reduced cytokine release relative to an analogous CD3-targeted TCE (126). The same logic is now being tested clinically with AB248, a CD8β-targeted, affinity-attenuated IL-2 mutein (127), which is currently being evaluated in combination with the DLL3×CD3 HLE BiTE^®^ molecule (tarlatamab) in the phase 1b DeLLphi-311 study (NCT07037758). With CD3 TCEs, gated IL-2 delivery may therefore strengthen engager-primed effector function while reducing the liabilities of systemic cytokine exposure. CAR-T studies support the same principle. Orthogonal IL-2 systems pair an engineered IL-2 with a matched engineered IL-2Rβ to confine cytokine signaling to therapeutic T cells. In preclinical CAR-T models, orthogonal IL-2 and related IL-2-mutein strategies improved expansion, persistence, stem cell memory features, and tumor control (128). IL-15 provides the complementary persistence-oriented arm of Signal 3. In TCEs, CD3-bispecific exposure increases CD122 on activated T cells, supporting timed IL-15 delivery after engager priming. Accordingly, the IL-15/IL-15Rα-Fc fusion XmAb24306 enhanced T-cell expansion and converted transient anti-HER2/CD3 responses into durable complete responses in preclinical models (116). This strategy is now in phase Ib testing with the FcRH5×CD3 bispecific cevostamab (NCT05646836). In CAR-T cells, IL-15 preserved a stem-cell-memory-like phenotype, restrained mTORC1 activity, improved metabolic fitness, and enhanced *in vivo* efficacy, directly linking improved function and persistence to metabolic regulation (105, 129).

Metabolically, IL-2 amplifies the biosynthetic program initiated by Signal 1 by activating mTORC1-linked anabolic and glycolytic pathways in cytotoxic T cells, sustaining nutrient uptake, protein synthesis, cytokine production, and cytolytic effector-molecules (130, 131). In contrast, IL-15 signaling is more consistently associated with restrained mTORC1 activity, increased mitochondrial biogenesis, and respiratory reserve, supporting a memory/stem-like state and improving persistence of CAR T cells (105, 132–135). These distinct metabolic programs likely explain why combinations with IL-2 can amplify effector output, whereas IL-15 is better suited to sustain long-term memory, fitness and persistence in T-cell therapies (116, 118, 130, 131, 133). IL-2 and IL-15 induce distinct programs, highlighting cytokine signaling as a tunable regulator of TCE durability rather than simply a growth cue. Engineered cytokines and muteins may build on this by directing T cells toward fitter, more persistent metabolic states, an idea already supported by cytokine-armored or cytokine-supported TCE and CAR T-cell strategies (136–139).

## Signal 4 – environmental control of T cell immunity by nutrients, metabolic stress, and environmental sensing pathways

**Figure 2:**
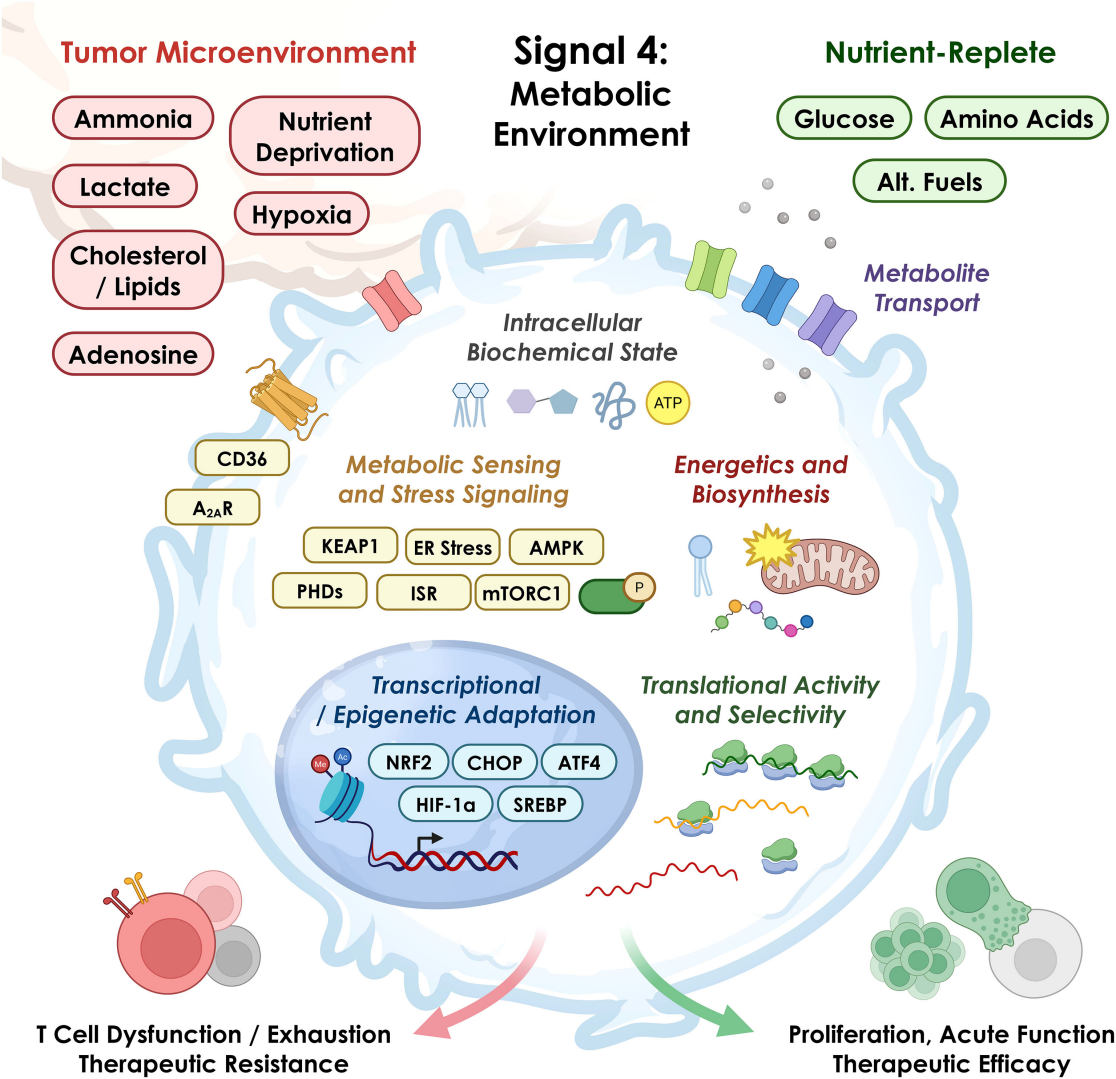
The biochemical environment acts as “Signal 4”, shaping T cell responses by governing metabolic activity, nutrient- and stress-sensing, and gene regulation. While replete environments contain ample nutrients to support protective immunity, tumor microenvironment displays a variety of biochemical perturbations that dampen T cell responses, potentially limiting TCE efficacy. Intracellular changes in biochemical substrates directly influence metabolic activities while also signaling to diverse nutrient-sensing and stress-signaling pathways. In combination, these acute responses drive transcriptional, translational, and epigenetic adaptations that intersect with immunological programming to influence acute T cell function and long-term fate. Understanding how biochemical environment, metabolic activity, and gene-regulatory responses influence T cell immune responses could help illuminate mechanisms of TCE efficacy or resistance in solid tumors. Created in BioRender. Engavale, M. (2026) https://BioRender.com/6312gh2.

Metabolic dysregulation is considered a defining hallmark of cancer, driven by both tumor-cell-intrinsic metabolic programming and environmental aberrations local to the tumor microenvironment (140–142). Indeed, immunosuppressive factors such as decreased nutrient availability, hypoxia, metabolic waste, and competition among tumor and immune cell populations within the TME represent key barriers for the success of immunotherapeutics in solid tumor indications (143, 144). In appreciation of this, environmental metabolites can be considered as “Signal 4” within modern models of lymphocyte function (**Figure 2**), emphasizing how biochemical cues work alongside classical surface receptor signals to shape T cell fate, function, and immunotherapeutic response (24, 145).

Nutrients are important positive regulators of T cell immunity, and nutrient acquisition and allocation are key requirements for discrete immunological functions like cytokine secretion, cytotoxicity, and proliferation (146). Glucose is perhaps the best-characterized regulator of T cell responses, influencing T cell biology through bioenergetic, gene-regulatory, and signaling mechanisms (147). Of note, glucose metabolism acutely regulates cytokine production and T cell proliferation (148–150), selectively regulates the translation of cytokine mRNAs such as *Ifng (*151, 152), governs cell cycle progression via acute expression of c-Myc (29, 153), and shapes global histone acetylation patterns (154, 155), making it a key target in adoptive T cell therapies (156–159). Glutamine and glutamate are also key substrates supporting T cell energetics and biosynthesis and influence Th1, Th17, and Treg differentiation by regulating histone and DNA methylation (38, 160–167). Beyond these key nutrients, other amino acids including branched-chain amino acids (leucine, isoleucine, and valine) (35), arginine (168–170), methionine (171–173), serine (37, 174), alanine (30), asparagine (175–177), and aspartate (178–183) have all been shown to regulate T cell activation or function, acting as key substrates for nucleotide synthesis, antioxidant generation, and protein translation. Additionally, beyond glucose or glutamine, lesser-appreciated carbon sources such as inosine (184, 185), acetate (57, 154, 160, 186, 187), and beta-hydroxybuturate (179–183) also support endogenous and therapeutic-driven T cell responses, reflecting the plasticity of T cell fuel choice.

On the other hand, nutrient deprivation in the TME, combined with hypoxia (77, 78, 188–196), metabolic wastes like lactate (181, 197–204) and ammonia (205–209), and immunosuppressive metabolic signals like extracellular adenosine (190, 210–212), cholesterol (213), and excess fatty acids (214) impede the anti-tumor T cell response and remain major challenges for immunotherapy. In many cases, these environmental perturbations directly act on effector T cells, acutely limiting T cell function and driving exhaustion – for example, by suppressing glycolysis or driving the expression of immune checkpoint receptors such as PD-1. In other cases, tumor cells, myeloid cells, or cancer-associated fibroblasts actively influence the shared immunometabolic environment by secreting or sequestering metabolites that could restrict effector T cell activity or promote immunosuppressive immune cell polarization, such as regulatory T cells or macrophages. Given that TCE responses in solid tumor models rely on engaging tumor-infiltrating T cells (45), intra-tumoral metabolic crosstalk could prove particularly important to fully understanding TCE efficacy or resistance and support therapeutic hypotheses targeting additional cell types within the TME that interact with T cells.

Beyond impacting cell biochemistry, nutrients and environmental stressors are actively sensed by a suite of signaling pathways, including the mammalian target of rapamycin (mTOR) (35, 38), AMP-activated protein kinase (AMPK) (151, 215–217), integrated stress response (ISR) (193, 218–222), ER-stress response (80, 213, 223–229), hypoxia-inducible factor (HIF) (77, 188, 189, 192, 196), and nuclear factor erythroid 2-related factor 2 (NRF2) signaling axes (176, 230–232). These pathways act as an additional feedback mechanism on immune function, jointly controlling gene regulatory pathways, metabolism, and immune programming alongside classical surface receptor signals. While chronic stresses repress effector functions and potentiate T cell exhaustion via factors such as ATF4, CHOP, or HIF-1a (77, 78, 189, 193), transient or mild stresses drive metabolic and regulatory adaptations that maintain or enhance T cell function despite suboptimal environmental conditions (221, 233, 234). While studies mechanistically exploring “Signal 4” during TCE responses is limited, evidence that blockade of adenosine signaling by anti-CD39/CD73 blocking antibodies or an A2A receptor antagonist enhances T cell effector function during TCE responses (235, 236) while hypoxia interferes with TCE efficacy in a manner dependent on VEGF secretion (237), suggesting that environmental regulation of T cell responses could be targeted concurrently with TCE therapy to improve response, analogous to previous efforts in the context of immune checkpoint blockade (238).

## Discussion

Current TCE designs rely on manipulating surface receptor signaling to shape T cell function, and next-generation approaches continue to vary molecular formats, affinities, and targeted signaling axes within this same paradigm (239). Despite encouraging progress, the additional biochemical challenges inherent to solid tumors require new design paradigms to overcome. By emphasizing metabolism as an integrator of immune signaling and environmental conditions during T cell responses, this 4-signal model offers a more holistic framework for understanding TCE-driven T cell function, fitness, and fate in solid tumors. The next challenge for the field is to refine this framework using direct evidence from TCE therapy itself, applying novel technologies for immunometabolic profiling to characterize preclinical disease models or on-treatment clinical samples (240–242). Once realized, a metabolically-informed model of TCE responses would open new opportunities to better understand TCE pharmacodynamics across solid tumor types, identify tumor-intrinsic, T cell-intrinsic, and microenvironmental biomarkers of response and resistance, and discover additional targetable axes for next-generation TCEs or combination immunotherapies.

Due to longstanding challenges of studying cell metabolism directly *in vivo*, little is known about which metabolites or pathways are most critical to sustain T cell responses during TCE therapy. With recent *in vivo* labeling and cell isolation techniques, metabolomics and stable isotope tracing could reveal how T cell nutrient acquisition and fuel use differs across TCE designs, between tissue locations, or over the duration of therapy (174, 243–246). Immune cells differ widely in their metabolic configurations and functional profiles; with careful validation, cytometry could be used to deconvolute subset-level contributions to overall TCE response, identify metabolic biomarkers for T cell function at single-cell resolution, or interrogate the metabolic configurations of other TME-resident cell types such as myeloid cells or fibroblasts (247–256). While the generalized TME described here is a useful heuristic, it will be important to define instances where metabolic perturbations show the strongest evidence of dampening TCE-driven immune responses (257). Comparative profiling efforts could be performed across solid tumor types, between primary or metastatic sites, or even within individual tumors. Here, single-cell and spatial methods for biochemical profiling could be integrated with gene expression data to capture both biochemical and genomic dimensions of the TME and support disease hypotheses, such as oncogenic drivers, cell-cell metabolic crosstalk, or disrupted tissue architecture (255, 258–262). While insights from patient samples remain indispensable, *in vitro* models could also utilize physiological or disease-relevant media to improve metabolic fidelity for TCE candidate profiling, or to build metabolically-informed PKPD models for predicting functional responses across TCE designs or environmental contexts (181, 263–269). As our understanding of TCE-driven metabolism evolves, perturbation by pharmacological (270) or genetic methods (271, 272) could build conviction towards directly targeting metabolic axes to improve TCE response or safety, or informing TCE candidate selection based on the metabolic profiles of responding cells. Lastly, TCEs could provide a valuable platform for understanding how metabolism and immunity broadly intersect, informing strategies to target T cell metabolism for therapeutic benefit beyond oncology indications, including allergy, autoimmune disease, and transplantation biology (273–278).
